# Valorization of Kraft Lignin from Black Liquor in the Production of Composite Materials with Poly(caprolactone) and Natural Stone Groundwood Fibers

**DOI:** 10.3390/polym14235178

**Published:** 2022-11-28

**Authors:** Quim Tarrés, Roberto Aguado, Juan Domínguez-Robles, Eneko Larrañeta, Marc Delgado-Aguilar

**Affiliations:** 1 LEPAMAP-PRODIS Research Group, University of Girona, Maria Aurèlia Capmany 61, 17003 Girona, Spain; 2 School of Pharmacy, Queen’s University Belfast, Lisburn Road 97, Belfast BT9 7BL, UK

**Keywords:** thermoplastic lignin, poly(caprolactone), blend, biocomposite, mechanical properties, waste exploitation

## Abstract

The development of new materials is currently focused on replacing fossil-based plastics with sustainable materials. Obtaining new bioplastics that are biodegradable and of the greenest possible origin could be a great alternative for the future. However, there are some limitations—such as price, physical properties, and mechanical properties—of these bioplastics. In this sense, the present work aims to explore the potential of lignin present in black liquor from paper pulp production as the main component of a new plastic matrix. For this purpose, we have studied the simple recovery of this lignin using acid precipitation, its thermoplastification with glycerin as a plasticizing agent, the production of blends with poly(caprolactone) (PCL), and finally the development of biocomposite materials reinforcing the blend of thermoplastic lignin and PCL with stone groundwood fibers (SGW). The results obtained show that thermoplastic lignin alone cannot be used as a bioplastic. However, its combination with PCL provided a tensile strength of, e.g., 5.24 MPa in the case of a 50 wt.% blend. In addition, when studying the properties of the composite materials, it was found that the tensile strength of a blend with 20 wt.% PCL increased from 1.7 to 11.2 MPa with 40 wt.% SGW. Finally, it was proven that through these biocomposites it is possible to obtain a correct fiber–blend interface.

## 1. Introduction

Research in the area of materials knowledge has classically been based on the development of new materials with better physical–mechanical properties than the materials used at that time. Among the most widely used materials today are plastics, which have great mechanical properties, low cost, durability, and a wide range of different types that allow the production of various products/applications used in daily life [1]. However, the environmental problems arising from the use of non-renewable and mostly fossil-based materials make their production unsustainable [2]. For this reason, research in recent years has focused on the development of bio-based materials and a closed-loop circular economy with maximum waste reduction, recycling, and reuse [3]. As a result of this research, various thermoplastic biopolymers are being used, such as polylactic acid, poly(butylene succinate), poly(3-hydroxybutyrate-co-3-hydroxy valerate), thermoplastic starch, or poly(caprolactone) (PCL) [4]. PCL is a polymer used for a wide variety of applications, especially in the biomedical field [5,6,7,8,9]. This polymer has received a lot of attention as it presents interesting properties for the development of a wide variety of materials. It is a biodegradable/biocompatible polymer that presents high strength and can withstand solvents, chlorine, oil, and water [7]. In addition to all these properties, PCL has a low melting point (ca. 60 °C) that makes it very simple to form blends with other compounds via melting. Moreover, due to its low melting temperature, multiple studies have reported the use of PCL for extrusion-based 3D printing [10,11,12,13,14,15]. The properties of PCL can be tailored by combining it with natural products such as lignin or natural fibers [7]. However, most of these bio-based plastics present high production costs that do not make them industrially competitive [16].

On the other hand, lignin is a naturally occurring and abundant polymer that constitutes between 15% and 40% (dry weight) of wood and most other lignocellulosic biomasses, providing rigidity to the cell walls [17,18,19,20]. It is characterized by its complex polyphenolic structure and its amorphous nature. Lignin is a highly branched biomacromolecule; due to the many significant functional groups such as hydroxyl, methoxyl, carboxyl, and carbonyl groups present it provides special functional properties such as oxidation resistance, stabilization, reinforcement, biodegradability, antifungal and antibiotic activity, or resistance to UV absorption. Moreover, lignin is available as a by-product in large quantities from the pulping industries and lignocellulosic biorefineries. The complex structure of this aromatic macromolecule can vary depending on its botanical origin and on the extraction procedure applied [21,22,23,24]. Usually, the lignin present in black liquor is not used but is sent directly to recovery boilers to generate energy [25]. For lignin valorization, lignin fractionation is currently considered to be a key step [26]. However, traditional solvents (such as dilute acid, alkaline solvents, and organic solvents) are considered to be unsuitable for lignin fractionation [27]. Although there are new methods of lignin valorization using bio-based organic solvents and ionic liquids, the complex synthesis procedures, high toxicity, or low recoverability may not be commercially profitable and restrict their practical applications [28,29]. However, one of the ways to add value to this by-product is its use for the manufacture of thermoplastic lignin-based materials. The modification of lignin by temperature and shear forces in the presence of a plasticizing agent allows the production of a thermoplastic polymer with low mechanical properties [30]. Plasticization of lignin is due to swelling in its structure caused by the plasticizer. The plasticizing agent increases the free volume by spacing the polymer chains and the mobility of the chain segments, resulting in a decrease in Tg and melt viscosity [31]. Lignin plasticization also contributes to improved lignin dispersion in polymeric matrices [32]. However, the addition of lignin for the development and manufacture of these composites may provide interesting features to them, such as antioxidant and antimicrobial properties [17,33,34], resistance to chemical and biological attacks [35], and fire retardance [34,36,37].

Due to its low mechanical properties, thermoplastic lignin is commonly used as a blend or composite with other bio-based and/or biodegradable thermoplastics [38,39]. The production of blends requires obtaining a homogeneous material from the mutual miscibility of both components that interact by interdiffusion [40]. Although lignin blends with many different polymers have been studied, in all of them, lignin is a minority component due to its mechanical properties. Its use as a water resistance agent in starch films has been studied [41]. Other authors have evaluated lignin blends with polyhydroxyalkanoates, where it was observed that the presence of lignin reduced the crystallinity of polyhydroxybutanoate (PHB) and increased its recyclability [35]. Lignin can also be used to improve the flexibility and degradation rate of polylactic acid [42,43] or to improve properties such as UV resistance based on poly(propylene carbonate) [44].

Composite materials, on the other hand, are those that present two or more differentiated phases, with the creation of an interface between them. Research in the field of composite materials mainly focused on the use of synthetic fibers has given way to the use of natural fibers with lower reinforcement capacity but greater sustainability [45]. The use of natural fibers reduces the amount of plastic used in the manufacture of the material, improves mechanical properties, and reduces costs [46,47]. However, at the same time, the use of these materials implies an increase in the density of the material. On the other hand, in most composite materials there is a difficult interaction between the plastic polymer and the natural fibers [48]. Presumably, this difficulty should not exist between lignin and natural fibers since lignin is present in natural fibers. However, the authors are not aware of any literature where thermoplastic lignin-based composites reinforced with natural fibers have been studied. Even so, in the bibliography, some works demonstrate the effectiveness of the presence of lignin as a promoter of bonds between natural fibers and plastics [49,50].

This work studies the use of the lignin present in the black liquor of a hardwood (eucalyptus) kraft cooking process for the production of plastic materials. For this purpose, the lignin obtained by incorporating different glycerin contents (from 10 to 40 wt.%) has been plasticized. Subsequently, the blending properties of the plasticized lignin with poly(caprolactone) (PCL) (from 10 to 30 wt.%) have been evaluated. Finally, natural fibers have been incorporated as reinforcement to the lignin and PCL blends.

## 2. Materials and Methods

### 2.1. Materials

Black liquor obtained from a kraft cooking process to obtain bleached eucalyptus hardwood fibers was kindly supplied by LECTA Group, S.A. PCL CAPA^TM^ 6500 was obtained from Perstorp Specialty Chemicals AB (Perstorp, Sweden). The PCL used has a melting point between 58 and 60 °C and a melt flow rate (MRF) of 7 g/10 min. The glycerin used for thermoplasticization of the lignin was obtained from Scharlau S.A. (Barcelona, Spain), with 95% purity and a density of 1.26 g/cm^3^. Stone groundwood (SGW) softwood fibers were kindly provided by Zubialde, S.A. (Aizarnazabal, Spain). All the reagents necessary to obtain the precipitated lignin and characterization of the materials were obtained from Scharlau S.A. at analysis grade and used without further purification.

### 2.2. Methods

**Figure 1:**
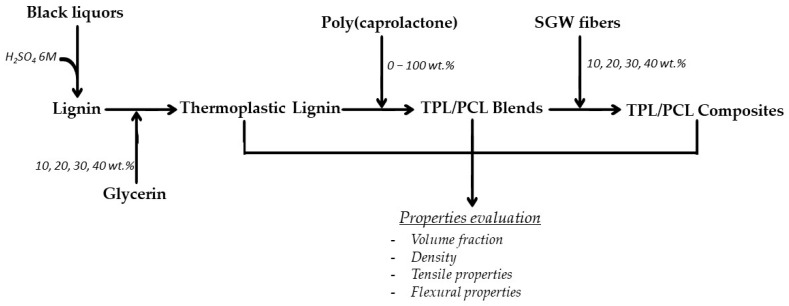
Flowchart diagram of the experimental procedure.

The flow chart shown in Figure 1 illustrates the experimental procedure performed for the study of lignin blends and compounds obtained from kraft black liquor.

#### 2.2.1. Precipitation and Thermoplasticization of Lignin

Precipitation of kraft lignin from black liquor was accomplished by lowering the pH from 12.45 to 2 by the addition of sulfuric acid, a procedure extensively reported in the literature [51,52] that also increases the material yield of the kraft pulp mill [53]. Lignin precipitation was performed on a volume of 10 L of black liquor by adding sulfuric acid with a concentration of 6 M. The acid was incorporated progressively in a system with constant agitation of 300 rpm. Once pH 2 was reached, the agitation was stopped and the suspension was kept at rest for 24 h for sedimentation of the precipitated lignin. Finally, the precipitated lignin was separated and washed by centrifugation and repeated addition of distilled water. The lignin obtained was dried at 90 °C until at constant weight.

The black liquor and the extracted lignin were both characterized. In both cases, the ash content was analyzed according to the TAPPI T211 standard. The content of extractives present was also evaluated via ethanol-benzene extraction according to TAPPI T204 cm-97 and the content of Klason lignin according to TAPPI T22 om-98 in the extractives-free sample. In the black liquor, the presence of total monosaccharides present was also evaluated following TAPPI T249. Additionally, the precipitated lignin was characterized by thermogravimetric analysis (TGA) and Fourier transform infrared spectroscopy (FTIR). A Mettler Toledo SDTA 851 thermobalance (Mettler Toledo, L’Hospitalet de Llobregat, Spain) was used for the TGA, working in a range of temperatures from 30 to 700 °C with a heating rate of 10 °C/min in an inert atmosphere (N_2_). FTIR analysis was performed by Matson Satellite FTIR and using the analysis software WinFirstTlite.

Once the lignin was obtained, it was plasticized with different percentages of glycerin (between 10 and 40 wt.%) using a Brabender W 50 EHT (Brabender GmbH & Co. KG, Duisburg, Germany). The conditions used for thermoplasticization of the lignin were in all cases 130 °C and 80 rpm for 13 min. Once the mixing time elapsed, the thermoplastic lignin (TPL) was removed from the equipment and cooled at room temperature. Once the thermoplastic lignin was solidified, it was pelletized in a knife mill (Restsch Cutting Mill SM-100, Retsch GmbH, Haan, Germany) and stored at a temperature of 80 °C until use to avoid moisture absorption.

#### 2.2.2. Development of Lignin and PCL Blends and Their Composites Reinforced with Natural Fibers

The preparation of the thermoplastic lignin and PCL blends was carried out by adding between 0 and 100 wt.% PCL at 10 wt.% intervals, as summarized in Table 1. For this purpose, the Brabender was used again at 130 °C and 80 rpm for 13 min. The blends obtained were pelleted and processed via injection after being previously dried for 24 h at 80 °C. Moreover, final composite materials were produced by adding between 10 and 40 wt.% of SGW fibers to the Brabender equipment. Initially, the blend of lignin and PCL was preheated to 130 °C. Once the blend was completely melted, the fibers were incorporated at 80 rpm for 10 min to ensure their correct dispersion. As with the previous materials, the composites obtained were pelletized and stored at 80 °C until processing.

Standard specimens were obtained by injection molding with an Arburg 220M 350-90U injection molding machine (Arburg, Lossburg, Germany). Specimens of 115 mm length with narrow grips, thickness 3.2 mm, and 13 mm gauge width were produced. The injection conditions were a temperature profile of 120, 130, 140, and 150 °C, with the first pressure of 120 kg/cm^2^ and the second pressure of 37.5 kg/cm^2^. The obtained composites were conditioned at 23 °C and 50% relative humidity for 48 h in a climatic chamber (Dycometal CCK-30/1000, Dycometal Inc., Viladecans, Spain) before characterization, following ASTM D618.

#### 2.2.3. Materials Characterization

The physical properties of the composite materials obtained were determined by characterizing their density. The density was determined using a solid pycnometer according to ISO 1183-3. By determining the densities of the composite and the matrix it is possible to calculate the volume fraction of the composites according to the expression

(1) $$V^{F}=\frac{w^{F}\cdot w^{m}}{w^{m}\cdot \rho^{F}+w^{F}\cdot \rho^{m}}$$

where $w^{F}$ and $w^{m}$ are the mass fractions of the natural fiber and plastic matrix used for composite production, $\rho^{m}$ the matrix density, and $\rho^{F}$ the fiber density calculated from the composite density $\rho^{C}$ obtained experimentally according to the following eq:

(2) $$\rho^{F}=\frac{\rho^{C}-\rho^{m}\cdot w^{m}}{w^{F}}$$

The characterization of the mechanical properties was carried out on standard specimens obtained by injection molding. All tests were performed on a minimum of 10 specimens.

The tensile test was used to determine the maximum tensile strength, Young’s modulus, and the tensile deformation of the material. In this sense, the test consisted of measuring the force required to deform the standardized specimens when they were subjected to stretching at a constant speed using a TM 1122 universal testing machine (Instron, United States). A load cell of 5 kN, a constant testing speed of 2 mm/min, a distance between grips of 11.5 cm, and an extensometer MFA2 (Metrotec, Spain) for more precise strain measurements according to ASTM D3039 were used for these tests.

As is widely known, the force required to deform a material depends, among other characteristics, on its dimensions. In this sense, the tensile strength is expressed as nominal stress (MPa), obtaining the tensile strength of the material without the influence of the section of the specimen measured (thickness and width) for each of the specimens tested. For the determination of Young’s modulus, values between 0.05 and 0.25% of deformation were used. Young’s modulus is used to evaluate the stiffness of the material or the difficulty of deforming a material.

On the other hand, the bending test allows for determining the bending strength, the elastic modulus in the elastic region, and the maximum bending deformation. As with the tensile test, it was performed using a TM 1122 universal testing machine (Instron, United States) at a constant speed of 2 mm/min according to ASTM D790.

The maximum flexural strength was calculated according to the following equation:

(3) $$\sigma_{F}=\frac{3\cdot F\cdot L}{2\cdot w\cdot h^{2}}$$

where $\sigma_{F}$ is the maximum bending strength, F the maximum test force, L the distance between the two support points, *w* the specimen width, and h the thickness.

The elastic modulus was obtained in the elastic region of the test curve according to the following equation:

(4) $$E_{F}=\frac{L^{3}\cdot F}{4\cdot w\cdot h^{3}\cdot \delta }$$

where $E_{F}$ is the elastic modulus and δ is the deflection of the standard specimen.

## 3. Results and Discussion

### 3.1. Black Liquor and Lignin Characterization

The main characteristics of the black liquor as well as the chemical composition and density of the extracted lignin are presented in Table 2.

The black liquor, with a pH of 12.45, had a density of 1.176 g/mL. Values of pH higher than 10 are usual for black liquor coming from kraft-type digestion, as it is well-known that kraft processes are carried out by means of a strong alkaline solution (pH~14) [54,55]. On the other hand, the density presented by the black liquor is slightly higher than the values reported by Cardoso et al. [56], very close to 1 g/mL. Moreover, other authors reported similar black liquor density values for black liquor coming from eucalyptus kraft cooks [57,58]. The solids content contained in the black liquor was 28.8 g/L, a similar value to those reported in the literature, which is between 25 and 35 g/L [58,59]. Precipitation of kraft lignin from black liquor was performed by decreasing the pH from 12.45 to 2 by adding 290 mL of 6 M sulfuric acid per liter of black liquor. The amount of lignin precipitated depends largely on the acid used for its precipitation, the acid concentration, the final pH, and the addition rate, as reported by Domínguez-Robles et al. [59]. In this study, by using the conditions defined previously, 15.3 g of lignin were obtained per liter of black liquor.

On the other hand, the purity and chemical composition of the resulting lignin is directly related to the type of lignocellulosic biomass used as raw material, the cooking process and its conditions, and the precipitation and/or purification method applied. The extracted lignin fraction in this study exhibited a relatively low Klason lignin content (44.72%), as well as relatively high ash content (13.12%). Kraft pulping is well-known for producing significant lignin depolymerization due to its severe cooking conditions [60,61]. This may explain the lower value obtained for Klason lignin. The acid-soluble lignin (ASL) content is undoubtedly high. ASL is mainly composed of low-molecular weight degradation products and hydrophilic derivatives of this aromatic polymer [60]. Indeed, reaching such low pH values (pH 2 was reached in this study) can also lead to the presence of lignin with a lower molecular weight [26]. Additionally, the elevated ash content found in the extracted lignin sample may be explained by the NaOH used in the kraft process, which seems to contribute the bulk of the ash. Moreover, lignin obtained in this study possessed a density of 1.384 g/L.

**Figure 2:**
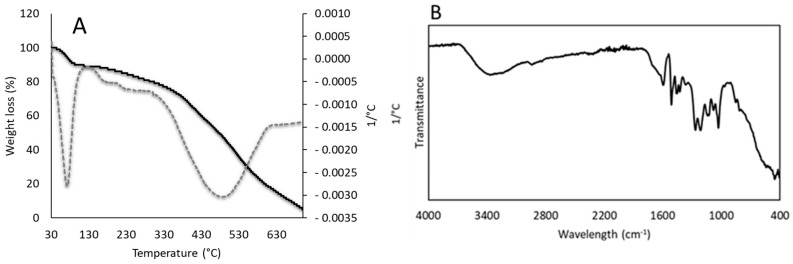
TGA and DTGA curves (**A**) and FTIR spectra (**B**) of the obtained kraft lignin.

Thermogravimetric analysis (TGA) of the lignin obtained (Figure 2A) was performed to determine the weight loss as a function of thermal degradation temperature (TG) as well as the weight loss ratio (DTG). The TG curve (solid line) shown in Figure 2A exhibited an initial weight loss between 25 and 100 °C, which is due to the water loss. After this step, the second stage in which the weight loss became more apparent was between 150 °C and 400 °C. This weight loss can be attributed to the lignin content [62]. Moreover, the broad range of temperature is due to the complexity of its structure. The weight loss in this second stage started slightly earlier (150 °C) in comparison with the values reported in the literature for other types of lignin samples (200–230 °C) [60,63]. These differences can be explained by the lower percentage of Klason lignin (44.72%), and thus most likely the high contents of ASL and/or carbohydrates provided a lower T_onset_ value for the extracted sample, which also corroborates the above discussion. Finally, the degradation experienced above 400 °C is attributed to the decomposition of aromatic rings [62,64]. The content of residues present after thermogravimetric analysis was less than 10%. Additionally, the DTG curve (dashed line) in Figure 2A clearly shows the three degradation peaks mentioned above. The DTG analysis presented unusual peaks at about 400 °C. This could be a consequence of the impurities present in the sample due to the non-post-treatment of the sample after precipitation.

Fourier transform infrared (FTIR) analysis of lignin obtained in the region of 4000 to 400 cm^−1^ is shown in Figure 2B. The lignin sample exhibited a characteristic broad peak within the range between 3600 and 3400 cm^−1^ corresponding to both aromatic and aliphatic hydroxyl groups present in lignin. The band observed at around 2900 cm^−1^ may be indicating the presence of methyl and methylene groups. Bands located at 1600, 1510, and 1420 cm^−1^ can be attributed to the vibration of the aromatic rings of the lignin. Moreover, the band located at 1450 cm^−1^ is assigned to the asymmetric deformation of C-H stretching [51]. Additionally, more bands were identified in the kraft lignin spectra. For instance, a band located at 1210 cm^−1^ can be related to the vibrations of guaiacyl rings. On the other hand, the band located at 1030 cm^−1^ is associated with the deformation vibrations of C-H bonds in the aromatic rings and deformation vibrations of C-O bonds in primary alcohols [51,60]. The bands located at 830 and 780 cm^−1^ are related to the vibration of the C-H bonds in the aromatic rings. Finally, the peak observed at around 615 cm^−1^ can be attributed to the presence of C-S bonds coming from the sulfuric acid used for lignin precipitation [59].

### 3.2. Thermoplastic Lignin Characterization

Table 3 shows the glycerin contents used to produce different thermoplastic lignins, the volume fraction of glycerin, and the density of the resulting thermoplastic lignin.

**Figure 3:**
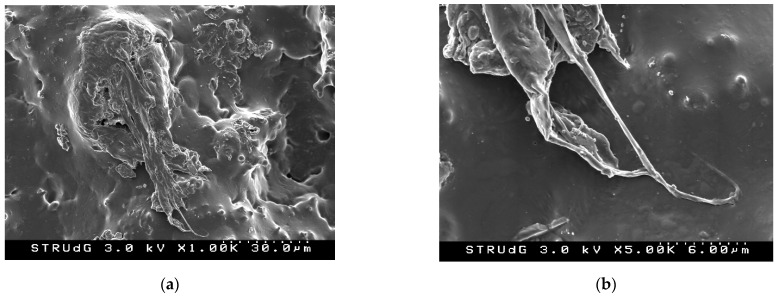
Scanning electron microscopy images of 90% lignin + 10% glycerin thermoplastic lignin: (**a**) magnification at 30 µm; (**b**) magnification at 6 µm.

As shown in Table 3, all the lignin obtained had a density higher than 1.3 g/mL. This means that the production of bioplastics from lignin represents a notable increase in density concerning fossil plastics with densities usually below 1 g/mL. On the other hand, as expected, the incorporation of a higher glycerin content with a density lower than that of lignin causes a gradual decrease in the density of thermoplastic lignin. In this sense, the plasticizing effect of the glycerin is corroborated, which together with the shear forces and the temperature increase produced during the mixing in the plastograph generated the modification of the lignin structure, incorporating the glycerin and giving rise to a continuous material as was observed by means of the SEM images presented in Figure 3.

In the images in Figure 3 it can be seen how the plasticization of the lignin has been carried out, effectively resulting in a uniform material. However, some unplasticized lignin granules can be observed after this first pass of the material through the Brabender. No significant differences were observed between the microscopy images for the different percentages of glycerin studied, demonstrating that low glycerin content allows the lignin to be thermoplasticized correctly.

Table 4 shows the tensile strength, Young’s modulus, and elongation at break as well as flexural strength, flexural modulus, and elongation results for the different thermoplastic lignins obtained.

The thermoplastic lignin samples with 40 wt.% glycerin could not be tested due to their brittleness. On the other hand, as can be seen in Table 4, the tensile strength of the thermoplastic lignin obtained is practically 0. As can be seen, the higher presence of glycerin decreases the tensile strength, although the differences cannot be considered significant due to the low values presented and their standard deviations. On the other hand, the presence of a higher plasticizing agent (glycerin) leads to a decrease in Young’s modulus. It is known that the presence of a plasticizing agent allows us to decrease the stiffness of the material, but at the same time an excess of it can cause phase separation. In this case, free glycerol is present, and the intermolecular interactions are so low that the lignin simply slides away [65,66]. In the same sense, bending properties decrease with increasing glycerin content.

Therefore, although lignin alone cannot be considered for use as a bioplastic, its characteristics of compatibility with cellulose, antimicrobial properties, and bio-based and biodegradable character make it very interesting for its combination as a blend with plastics and as a compatibilizer for the incorporation of natural fibers.

### 3.3. Blends

Thermoplastic lignin and PCL were combined and analyzed. The results of the blends between the three types of thermoplastic lignin obtained and PCL with additions between 0 and 100 wt.% are presented below. As shown in Table 5, the higher presence of glycerin in the thermoplastic lignin and therefore its lower density caused a slight difference in the volume fraction of PCL added for the same weight addition. This, however, does not represent a significant difference in the density of the resulting mixture for the same weight addition. However, this difference in the volume fraction present in the different blends must be considered for the assessment of their mechanical properties.

On the other hand, the lower density of PCL led to a substantial decrease in blend density when the PCL was increased.

**Figure 4:**
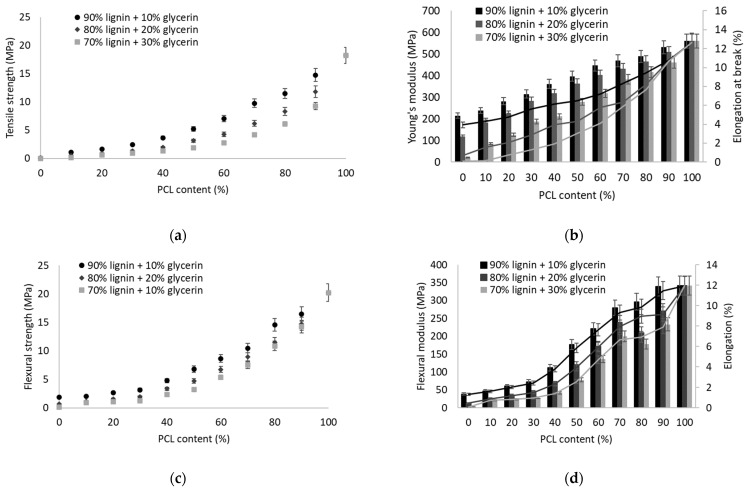
Mechanical properties of different TPL/PCL blends: (**a**) tensile strength; (**b**) Young’s modulus and elongation at break; (**c**) flexural strength; (**d**) flexural modulus and elongation.

Figure 4 shows the mechanical properties of the different blends obtained. Traditionally, lignin or thermoplastic lignin has been incorporated in low proportions into plastic materials to give them special properties [67,68]. In this case, the whole spectrum of possibilities was evaluated to use as much of this recovered material as possible. Although studies can be found in the literature showing that the incorporation of lignin in biodegradable plastic matrices [69], in most cases causes a decrease in mechanical strength [70].

The comparative values for the tensile strength, Young’s modulus, and elongation at break of the different blend compositions are reported in Figure 4a,b. In general, it is observed that the incorporation of low PCL content has a significant impact on tensile strength. The addition of 10 wt.% PCL increases the tensile strength from 0.112 to 1.122 MPa in the case of thermoplastic lignin with 10% glycerin. In the graph in Figure 3a, it can be seen how in the three cases of lignin with 10, 20, and 30 wt.% glycerin, the evolution in the tensile strength of the blend presents two different slopes. The two slopes can be divided between 0–40 wt.% and 50–100 wt.% of PCL; this is because the higher presence of PCL constitutes the thermoplastic lignin as a discontinuous phase and not as a matrix. The difference in properties between the different lignins evaluated is also remarkable. As observed, the presence of a lower glycerol content in the thermoplastic lignin leads to a blend with higher properties. This fact seems to indicate that the suitable glycerin content for the manufacture of thermoplastic lignin is around 10 wt.%. In the same sense, the incorporation of a higher PCL content with Young’s modulus value of 560 MPa compared to 215 MPa of lignin with 10 wt.% glycerin generates a progressive increase in Young’s modulus as well as in elongation at break. Although the incorporation of PCL permits obtaining a notable improvement in tensile properties, the blend obtained with 50 wt.% only presents 5.24 MPa of tensile strength, with Young’s modulus of 0.4 GPa and an elongation at break of 6.5%. If we compare these results with previous works with other biopolymers such as thermoplastic starch, we can see how they are lower than the 12 MPa of a blend of thermoplastic starch with 50 wt.% poly (butylene adipate-co-terephthalate) [71].

As for the flexural strength of the different blends, as shown in Figure 4c, the trend observed is similar to that presented in the tensile strength. However, in this case there is a smaller difference in behavior between the different thermoplastic lignins. Therefore, the glycerin content does not have a relevant effect on the flexural strength of the blend obtained. In general terms, a blend with 50 wt.% presented a bending strength of 6.8 MPa, higher than a thermoplastic starch that can present a bending strength of about 2 MPa [72] but still much lower than the more than 20 MPa of polyethylene [73]. As for the flexural modulus and deformation (Figure 4d), it can be observed that both increase with increasing PCL content, as expected.

### 3.4. Composites

Given the results obtained and to use of as much recovered lignin as possible, it was decided to continue with the incorporation of fibers using the blend with 20 wt.% PCL and the thermoplastic lignin prepared with 10% glycerin. The results of the study of the manufacture of TPL/PCL blend composites reinforced with natural fibers are presented below.

Table 6 shows the volume fraction of reinforcement for each composite, the density of the resulting composite, and the evolution of the fiber dimensions inside the composite as a function of the amount of composite.

One of the main limitations when incorporating natural fibers in a polymeric matrix is the modification of density. It is known that natural fibers with a higher density than most polymers of fossil origin, between 1.35 and 1.45 g/cm^3^, cause an increase in the density of the composite material by increasing the reinforcement content [74,75]. However, in this study the blend used as a matrix for the composite material presents a density similar to that of the fibers and therefore the density remains practically constant. In this sense, the incorporation of natural fibers as reinforcement of a TPL/PCL matrix is not limited by the increase in weight.

On the other hand, as seen in previous works, the increase in the volume fraction of reinforcement causes a greater generation of shear forces during the processing of the material, both in the kinetic mixer and in the injection of the piece, which causes a decrease in the length of the fiber and its diameter [75]. The fibers used initially have an arithmetic length of 259 µm, a weight-weighted length of 1659 µm, and a diameter of 34.1 µm, resulting in an aspect ratio of 7.6. The further reduction in the length but not in the diameter of the fibers due to processing of the composite material results in a decrease in the aspect ratio as the fiber content increases, a factor that must be taken into account for the development of the mechanical properties of the composite material.

Table 7 shows the results of the tensile and flexural properties of the composite materials. As can be observed, the evolution of Young’s modulus presented in Table 7 is linear. This indicates a correct distribution of the fibers in the material itself since the increase in Young’s modulus as a function of the amount of reinforcement is not a function of the quality of the fiber–matrix interface [76,77]. On the other hand, the increase in Young’s modulus also represented a notable decrease in the elongation at the break of the material.

The tensile strength of the composites increased significantly with increasing fiber content, reaching 11.2 MPa with 40 wt.% of fibers. This increase indicates that the interaction between fibers and blend is correct. However, to evaluate the theoretical strength of the interface, the simplest micromechanical model, known as the modified rule of mixtures (Equation (5)) was applied:

(5) $$\sigma_{t}^{C}=f_{c}\times \sigma_{t}^{F}\cdot V^{F}+\left(1-V^{F}\right)\times \sigma_{t}^{m^{*}},$$

The modified mixture rule indicates that the tensile strength of the composite depends, in a simplified form, on three factors: (i) the intrinsic properties of the fibers, represented by the intrinsic fiber strength ($\sigma_{t}^{F}$) and the volume fraction of fibers in the composite ($V^{F}$); (ii) the intrinsic properties of the matrix, represented by the matrix strength at the point of maximum deformation of the composite material ($\sigma_{t}^{m^{*}}$); and (iii) the efficiency factor ($f_{c}$). However, the equation has two unknowns, and therefore, to calculate the efficiency factor, it is necessary to know the intrinsic fiber strength. In the case of short fibers, the impossibility of experimentally determining the intrinsic resistance of the fibers through a single fiber test implies the application of different assumptions.

As a first assumption, the intrinsic strength of stone groundwood fibers is estimated to be about 250 MPa, as reported in the literature for polypropylene composite materials reinforced with these fibers without a coupling agent [78]. On the other hand, in the second assumption, an efficiency factor value for a compound with a strong interface, estimated at 0.18, was considered. The results obtained in both assumptions are shown in Table 8 [79].

**Figure 5:**
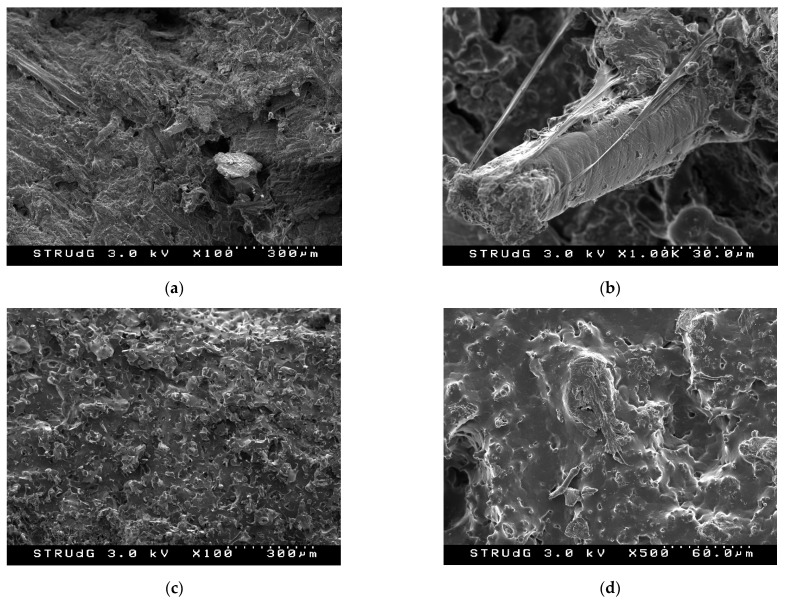
Scanning electron microscopy images of: (**a**,**b**) TPL/PCL blend reinforced with 30 wt.% SGW fibers, and (**c**,**d**) TPL/PCL blend.

In the initial assumption, the application of the rule of mixtures considering an intrinsic resistance of the fibers resulted in an efficiency factor in all cases close to 0.1, while in the second assumption considering a strong interface value the intrinsic resistance obtained was approximately 145 MPa. These results seem to indicate that the incorporation of stone groundwood fibers in a matrix consisting of a blend of thermoplastic lignin and PCL—although not ideal—is not weak, and therefore there is a certain level of anchorage of the fibers. This phenomenon was corroborated by scanning electron microscopy, as shown in Figure 5.

In the images of Figure 5a,b, taken in the fracture zone cryogenically treated with liquid nitrogen, it can be seen how the fibers are bonded to the matrix and how they have not slipped when subjected to stress. On the contrary, in Figure 5c,d, the blend without fibers is seen where the presence of the two polymers can be observed with relative cocontinuity. The achievement of high cocontinuity is reflected in improved mechanical properties due to the correct transfer of stresses between the two polymers. It can therefore be assumed that the interaction between the lignin present as the major component of the matrix and the fiber surface with a high lignin content (approximately 75% [80]) allows correct interphase.

The addition of SGW fibers and TPL to lignin has mainly been evaluated in this work by focusing on the mechanical properties. However, it is important to take into account that additives could bring added properties to the final material. In this case, the presence of lignin in the final material could provide antimicrobial and antioxidant properties to the final material [36,81,82,83,84]. This will contribute added value to the material.

## 4. Conclusions

This work has demonstrated the feasibility of using the lignin present in the black liquor of kraft digestion for the preparation of a biopolymer.

By precipitation at pH2 with 6M sulfuric acid, a high level of lignin recovery of 15.3 g/L in the black liquor was achieved. The chemical composition of the lignin obtained showed a high amount of extractives, which indicates that the lignin obtained has impurities due to the extraction process.

Through the study of obtaining lignin with glycerin as a plasticizing agent, the low mechanical properties of this material were observed, as well as how these properties decrease when the plasticizer content is increased. These properties were significantly improved by incorporating PCL for the production of a blend. The thermoplastic lignin with 10% glycerin showed the greatest aptitude to increase its properties, although it was observed that at high PCL contents the mechanical properties improved. However, it was found that to obtain acceptable mechanical properties using the highest amount of lignin, it is necessary to incorporate a reinforcement. The production of composite materials from a blend with 80% thermoplastic lignin demonstrated the possibility of obtaining lignin-based biocomposites with acceptable mechanical properties. In this sense, the simplified study of the micromechanics of the composites allowed the generation of a correct interface between the stone groundwood fibers and the TPL/PCL blend. Precipitated lignin has a brown coloration and, when thermoplasticized, it becomes darker, while composite materials have a black coloration. These materials do not present properties that allow them to be used in structural applications but they could be used in semi-structural ones, such as electrical household appliance casings, decorative parts in the automotive industry, etc.

## Figures and Tables

**Table 1:** TPL and PCL blends and SGW composite compositions.

Thermoplastic Lignin and Poly(Caprolactone) Blends			
Sample	Thermoplastic Lignin Content (wt.%)	PCL Content (wt.%)	SGW Content (wt.%)
TPL	100	0	-
90TPL/10PCL	90	10	-
80TPL/20PCL	80	20	-
70TPL/30PCL	70	30	-
60TPL/40PCL	60	40	-
50TPL/50PCL	50	50	-
40TPL/60PCL	40	60	-
30TPL/70PCL	30	70	-
20TPL/80PCL	20	80	-
10TPL/90PCL	10	90	-
PCL	0	100	-
**Composites with SGW**			
**Sample**	**TPL/PCL Blend Content** **(wt.%)**		**SGW Content** **(wt.%)**
80TPL/20PCL + 10SGW	90		10
80TPL/20PCL + 20SGW	80		20
80TPL/20PCL + 30SGW	70		30
80TPL/20PCL + 40SGW	60		40

**Table 2:** Black liquor physical data and lignin chemical composition.

Black Liquor			
Density (g/mL)	Total Dry Solids (g/L)	Acid spent (mL/L BL)	Lignin Extraction (g/L)
1.176	28.8	290	15.3
**Lignin**			
**Density** **(g/mL)**	**Ash** **(%)**	**Klason Lignin** **(%)**	**Acid-Soluble Lignin** **(%)**
1.384	13.12	44.72	6.93

**Table 3:** Glycerin conditions, the volume fraction of glycerin (V^G^), and density of the resulting thermoplastic lignin.

Glycerin (wt.%)	V^G^	Density (g/mL)
10	0.109	1.37 ± 0.01
20	0.215	1.36 ± 0.01
30	0.320	1.34 ± 0.02
40	0.423	1.33 ± 0.01

**Table 4:** Mechanical properties of different thermoplastic lignins (tensile and flexural).

Glycerin (wt.%)	Tensile Properties			Flexural Properties		
	Tensile Strength (MPa)	Young’s Modulus (MPa)	Elongation at Break (%)	Flexural Strength (MPa)	Flexural Modulus (MPa)	Elongation (%)
10	0.112 ± 0.536	215 ± 36	3.945 ± 1.126	1.874 ± 0.784	39 ± 12	1.322 ± 0.742
20	0.084 ± 0.697	119 ± 21	0.719 ± 0.542	0.682 ± 0.587	14 ± 16	0.466 ± 0.312
30	0.035 ± 0.342	21 ± 19	0.077 ± 0.345	0.123 ± 0.421	2 ± 7	0.081 ± 0.154
40	-	-	-	-	-	-

**Table 5:** Physical properties of thermoplastic lignin and PCL blends (TPL/PCL).

Thermoplastic Lignin	PCL (wt.%)	V^PCL^	Density (g/mL)
90% lignin + 10% glycerin	10	0.108	1.36 ± 0.01
	20	0.214	1.35 ± 0.01
	30	0.318	1.33 ± 0.01
	40	0.420	1.32 ± 0.01
	50	0.521	1.31 ± 0.02
	60	0.620	1.30 ± 0.01
	70	0.717	1.29 ± 0.01
	80	0.813	1.28 ± 0.01
	90	0.907	1.27 ± 0.02
	100	1.000	1.26 ± 0.01
80% lignin + 20% glycerin	10	0.107	1.36 ± 0.01
	20	0.212	1.34 ± 0.01
	30	0.316	1.33 ± 0.02
	40	0.418	1.32 ± 0.01
	50	0.518	1.31 ± 0.01
	60	0.617	1.30 ± 0.01
	70	0.715	1.29 ± 0.01
	80	0.811	1.27 ± 0.01
	90	0.906	1.27 ± 0.01
	100	1.000	1.26 ± 0.01
70% lignin + 30% glycerin	10	0.106	1.36 ± 0.01
	20	0.210	1.34 ± 0.01
	30	0.313	1.33 ± 0.01
	40	0.415	1.32 ± 0.01
	50	0.516	1.31 ± 0.01
	60	0.615	1.30 ± 0.01
	70	0.713	1.29 ± 0.01
	80	0.810	1.27 ± 0.01
	90	0.906	1.27 ± 0.01
	100	1.000	1.26 ± 0.01

**Table 6:** Physical properties of composites and morphological analysis of extracted fibers.

SGW Fibers (wt.%)	V^F^	Density (g/mL)	$l_{a}^{F}\;(µm)$	$l_{w}^{F}\;(µm)$	Width (µm)	Aspect Ratio ^1^
0	0.100	1.345 ± 0.06	-	-	-	-
10	0.100	1.345 ± 0.04	205	953	33.6	6.1
20	0.199	1.346 ± 0.07	195	859	33.4	5.8
30	0.299	1.346 ± 0.05	182	727	32.2	5.7
40	0.399	1.347 ± 0.05	172	627	30.3	5.7

^1^ Calculated as fiber length ($l_{a}^{F}$) divided by fiber width.

**Table 7:** Mechanical properties of composite materials of TPL/PCL and SGW.

SGW Fibers (wt.%)	Tensile Strength (MPa)	Young’s Modulus (MPa)	Elongation at Break (%)	Flexural Strength (MPa)	Flexural Modulus (MPa)	Elongation (%)
0	1.679 ± 0.543	282 ± 36	4.79 ± 1.05	2.669 ± 0.743	61 ± 12	2.048 ± 0.912
10	3.762 ± 0.875	642 ± 79	3.59 ± 0.87	8.008 ± 1.103	325 ± 34	1.568 ± 0.423
20	6.245 ± 0.674	1174 ± 46	2.32 ± 0.92	15.34 ± 1.546	529 ± 29	1.175 ± 0.687
30	8.269 ± 1.279	1567 ± 87	1.65 ± 0.63	22.47 ± 1.429	734 ± 41	1.010 ± 0.443
40	11.217 ± 1.875	1872 ± 64	1.24 ± 0.57	26.84 ± 2.061	1083 ± 63	0.880 ± 0.162

**Table 8:** Results of the application of the modified rule of mixtures under two assumptions.

SGW Fibers (wt.%)	$\sigma_{t}^{m^{*}}\;(\mathrm{MPa})$	First Assumption		Second Assumption	
		$\sigma_{t}^{F}\;(\mathrm{MPa})$	$f_{c}$	$\sigma_{t}^{F}\;(\mathrm{MPa})$	$f_{c}$
10	1.55	250	0.09	132	0.18
20	1.20	250	0.11	147	0.18
30	0.92	250	0.10	142	0.18
40	0.73	250	0.11	150	0.18

## Data Availability

Not applicable.
